# Using sulfur stable isotope ratios (δ^34^S) for animal geolocation: Estimating the delay mechanisms between diet ingestion and isotope incorporation in tail hair

**DOI:** 10.1002/rcm.9674

**Published:** 2023-11-28

**Authors:** Zabibu Kabalika, Daniel T. Haydon, Rona A. R. McGill, Juan M. Morales, Thomas A. Morrison, Jason Newton, J. Grant C. Hopcraft

**Affiliations:** ^1^ School of Biodiversity, One Health and Veterinary Medicine, Graham Kerr Building University of Glasgow Glasgow UK; ^2^ National Environmental Isotope Facility, Scottish Universities Environmental Research Centre University of Glasgow Glasgow UK

## Abstract

**Rationale:**

Metabolism and diet quality play an important role in determining delay mechanisms between an animal ingesting an element and depositing the associated isotope signal in tissue. While many isotope mixing models assume instantaneous reflection of diet in an animal– tissue, this is rarely the case. Here we use data from wildebeest to measure the lag time between ingestion of ^34^S and its detection in tail hair.

**Methods:**

We use time‐lagged regression analysis of δ^34^S data from GPS‐collared blue wildebeest from the Serengeti ecosystem in combination with δ^34^S isoscape data to estimate the lag time between an animal ingesting and depositing ^34^S in tail hair.

**Results:**

The best fitting regression model of δ^34^S in tail hair and an individual– position on the δ^34^S isoscape is generated assuming an average time delay of 78 days between ingestion and detection in tail hair. This suggests that sulfur may undergo multiple metabolic transitions before being deposited in tissue.

**Conclusion:**

Our findings help to unravel the underlying complexities associated with sulfur metabolism and are broadly consistent with results from other species. These findings will help to inform research aiming to apply the variation of δ^34^S in inert biological material for geolocation or understanding dietary changes, especially for fast moving migratory ungulates such as wildebeest.

## 1 INTRODUCTION

While the application of sulfur stable isotope ratios (δ^34^S) in ecological studies is not new (e.g. Peterson et al^1^), it has increased over the past two decades,^2^ mainly due to technological advances in mass spectrometry.^3^, ^4^ Specifically, the applicability of δ^34^S in reconstructing animal movement trajectories and diet shows promise for ecologists. For example, δ^34^S has been used in dietary studies about marine and marsh food webs,^5^ and δ^34^S in hair has been applied to study movement of animals in terrestrial^6^ and marine habitats.^7^ However, the delay between an animal ingesting and depositing sulfur in inert biological materials such as hair has rarely been explored or quantified, which limits the applicability of using δ^34^S for geolocation particularly for migratory animals.

Sulfur stable isotope ratios are generally considered to have small fractionation factor (i.e. diet–tissue difference in δ^34^S) during incorporation into both plant and animal tissues,^8^, ^9^, ^10^ and the δ^34^S values tend to vary with local geology.^2^, ^11^, ^12^ For instance, the reported fractionation factors for δ^34^S isotopes between diet and animal tissues are between −3‰ and +4‰^2^, ^10^, ^13^, ^14^, ^15^, ^16^ and between −8‰ and +4‰ between soil and plants,^8^ although there are some outlying values as high as +7‰ that have been reported.^10^ The small fractionation factor of δ^34^S makes it a good tracer of animal movement and diet in tissues because the values of δ^34^S in tissues stably reflect the δ^34^S in the local environment.

When applying stable isotopes to study animal movement, there are several requirements to meet and principles to consider.^17^, ^18^ For example, the first step is to have a tissue of interest from a consumer (e.g. muscle, feathers, blood or hair). This is because stable isotopes reflect the dietary history of organisms through their tissues.^19^, ^20^ The second is the time period of the tissue growth through which the spatial isotopic signature is retained. This is used to estimate the amount of movement information that can be studied over the tissue growth period. For instance, metabolically active tissues such as hair or feathers can provide a moving window of dietary information throughout the period of its growth,^21^, ^22^, ^23^ while an inactive tissue such as muscle or blood reflects a short period of its growth. One advantage of using metabolically inert biological material is that it can provide unique time‐series δ^34^S data from which animal movement and diet can be inferred.^22^, ^24^ This is because as animals move between different habitats, the information of past and present feeding is recorded and retained in these actively growing tissues, enabling scientists to infer movement history or diet change over time.^25^ Furthermore, biologically inert material is stable over long periods of time following synthesis, making it a useful archive of diet.^25^ Tail hairs are particularly interesting as longer lengths of hair can provide information over an extended period of time.^22^, ^23^, ^26^

However, the use of stable isotopes in animal tissues to infer movement requires the consideration of two important aspects. The first is establishing the diet–tissue discrimination factor which accounts for how the isotope value differs between tissue and diet.^27^, ^28^ The second is estimating the temporal lag between ingestion and the appearance of an isotopic change in animal tissue. Animal tissue does not immediately reflect the isotopic composition of diet, given that metabolism has an important role to play in determining the time before isotopic changes in diet and corresponding changes in tissues.^29^ This is because different elements are likely to be metabolised differently, and different tissues have different turnover and growth rates, so different delay effects can be expected.^29^ For example, sulfur is metabolised differently from carbon and nitrogen and across different tissues and species, because it occurs at low concentration in animals' tissues, and it is mostly bound within amino acids.^29^ However, some isotope mixing models (e.g. Stock et al^30^ and Parnell et al^31^) assume that the isotope composition of animal tissue is in short‐term equilibrium with diet (i.e. there is instantaneous reflection of diet in animals' tissue). This is not always the case and could mislead interpretation of isotopic results.^32^, ^33^, ^34^ Therefore, understanding the delay mechanisms associated with sulfur utilisation in inert biological materials is an important prerequisite to using the variation of δ^34^S to study different ecological processes.

In this study, we use δ^34^S data from GPS‐collared wildebeest from the Serengeti ecosystem to demonstrate the delay mechanisms involving incorporation of the δ^34^S isotope signal in tail hairs. We use GPS‐collared wildebeest to obtain the exact georeferenced location of animals in the landscape and compare the corresponding δ^34^S isoscape values against those values observed in the tail hair during the time of growth. We compare regression models between the landscape and the tail hair lagged over a period of up to 5 months. Our study provides insights into the processes behind δ^34^S signal delays in tail hair and helps to improve interpretation of δ^34^S results when making ecological inferences.

## 2 MATERIALS AND METHODS

**FIGURE 1:**
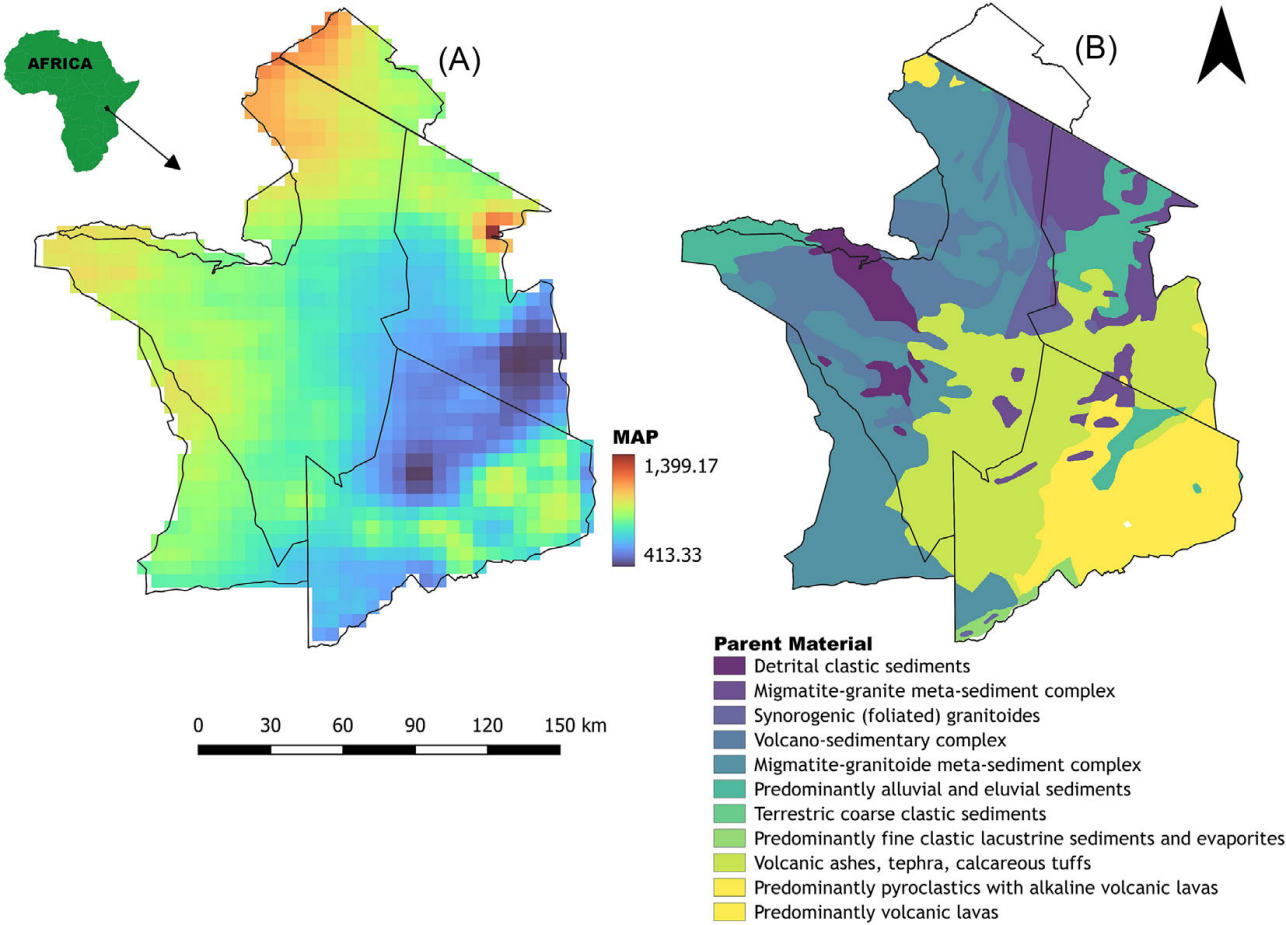
Map of Serengeti ecosystem showing (A) mean annual precipitation (MAP; data from CHIRPS repository: https://www.chc.ucsb.edu/data/chirps) and (B) the underlying parent material (data from Tanzania geological survey: https://www.gmis‐tanzania.com/). Protected area boundaries are shown in black solid lines. [Color figure can be viewed at wileyonlinelibrary.com]

**FIGURE 2:**
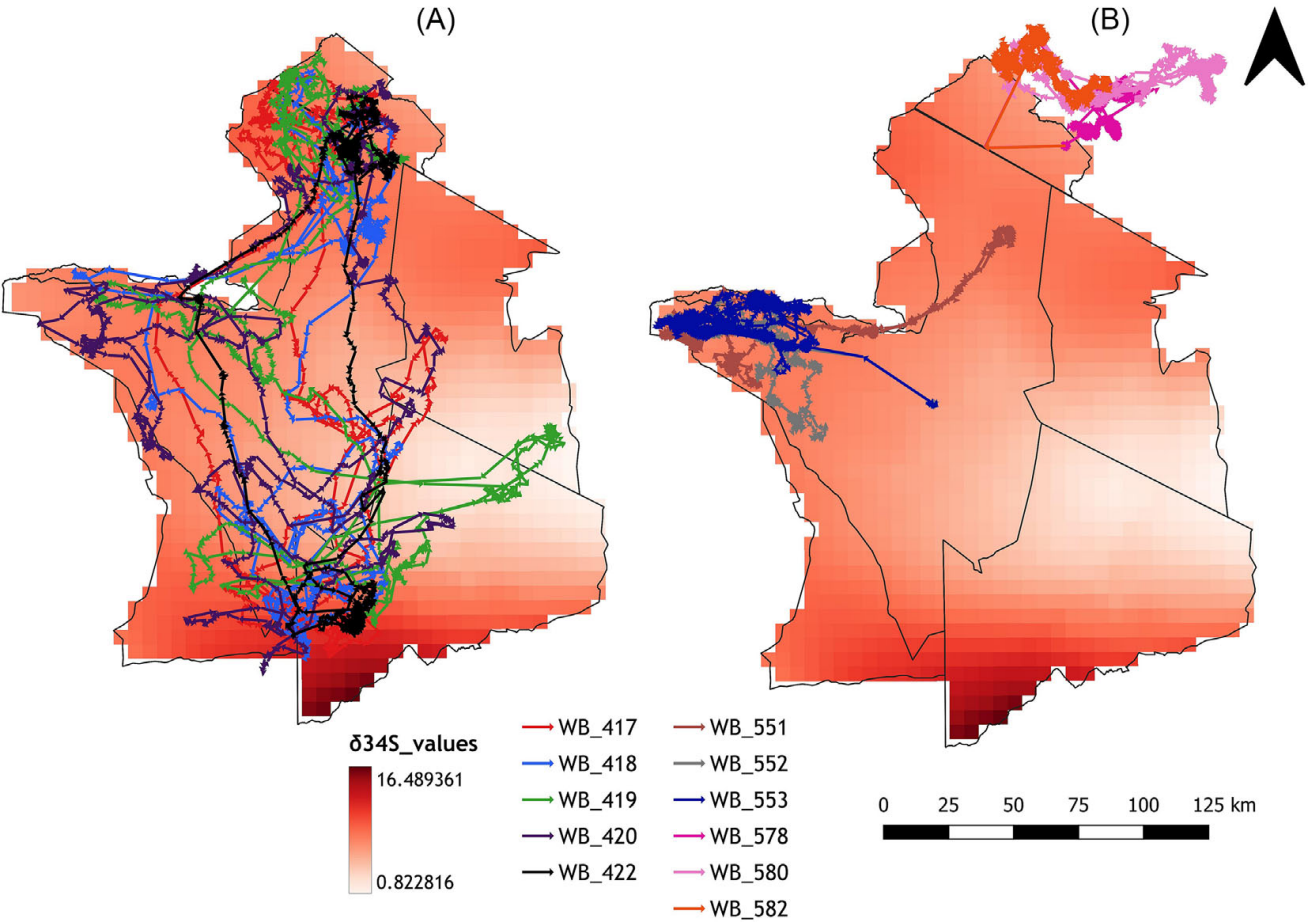
Variation of δ^34^S across the Serengeti ecosystem measured in the grass (data from Kabalika et al^6^). (A) Migratory route for the migratory wildebeest and (B) home range sizes for the resident wildebeest from both western corridor (WB 551, 552, 553) and Mara (WB 578, 580, 582). [Color figure can be viewed at wileyonlinelibrary.com]

Wildebeest tail hair samples were collected from the Serengeti‐Mara ecosystem in East Africa (Figure 1), between 34° and 36° E, and 1° and 3° N covering northern part of Tanzania and southern part of Kenya. The area is characterised by wet and dry seasons with rainfall of between 500 and 1200 mm per year (Figure 1A). Normally the dry season lasts for 5 months (June–October) and the wet season for 5 months (December–April) with November and May being transition months from dry to wet and vice versa, respectively.^35^ The ecosystem has a high gradient of soil fertility caused by heterogeneity of the underlying geology from young mineral‐rich pyroclastic material to ancient leached and eroded granite material^36^ (Figure 1B). These different soil types provide the ecosystem with a strong gradient of sulfur stable isotope ratios as reflected in the grass isoscape (Figure 2).^6^ The Serengeti grass sulfur isoscape ranges in δ^34^S values between −5‰ and 30‰ with measured δ^34^S values in grass ranging between +2.82‰ and +13.04‰.^6^ At any given site and any single time, δ^34^S values in grass have been characterised as having a standard deviation of _~_1.21 δ units (i.e. a 95% CI of about the mean ± 2.41 δ units).^6^

The ecosystem is home to 27 species of African ungulates including wildebeest which is the largest population of ungulates in the system (~1.3 million).^35^, ^36^ The wildebeest population is comprised of a mixture of migratory (~1.2 million) and resident individuals. Migrants move along a north–south trajectory (Figure 2A) which enables animals to capitalise on the grazing resources associated with the rainfall and soil fertility gradients.^36^

### 2.1 Collection and processing of biological materials

We collected tail hair samples from 11 GPS‐collared individual wildebeest (6 residents and 5 migratory individuals; Figure S1, supporting information). At the time of first capture, wildebeest were equipped with a GPS collar and the right side of their tail was shaved to skin level. The date, age, sex and reproductive status (i.e. whether pregnant or lactating) were recorded for each animal before it was released. After approximately a year, the collared animals were recaptured, and the regrown tail hair was collected along with the ancillary data as described above. The regrown tail hair from each animal was aligned and packed in paper envelopes pending laboratory analysis. The start and end dates of the sample allowed us to estimate tail hair growth rate, and the GPS data provided daily locations of the animal for the entire period of regrowth.

A small bundle of approximately 25 tail hairs from each individual were tied together so the proximal ends were aligned. Each bundle was prepared by washing in 2:1 chloroform–methanol and rinsed with double‐distilled water to remove the remnants of solvent.^6^, ^23^ Samples were dried for 48 h at room temperature. After drying, the total length of the tail hair was measured. This was to be used to calculate the tail hair growth rate. Tail hair samples were sectioned into 8 mm segments which correspond to approximately 2 weeks growth. The sectioning proceeded from the most recent part of the hair (proximal end) to the oldest (distal end). The segments were then powdered in a Retch MM400 (Germany) ball grinder using metal grinding tubes. The metal grinding tubes were immersed in liquid nitrogen for 60 s to embrittle the hair for easy powdering. The samples were ground for 90 s at 600 rpm. The powdered samples were weighed using a microbalance (Mettler Toledo, Model MX5, calibrated to three digits). Samples were weighed between 1.0 and 1.3 mg.

Samples were analysed for δ^34^S using a Pyrocube elemental analyser (Elementar Analysensysteme, Langenselbold, Germany) coupled to a VisION mass spectrometer (Elementar UK, Cheadle Hulme, Stockport, UK). The samples were analysed over three non‐consecutive runs spanning a 6 month period. Across these three runs we ran a number of laboratory and international standards as unknowns and obtained the following results. Laboratory standard ANR (powdered fish muscle), δ^34^S average value 18.335‰ (accepted value 18.81‰), standard deviation 0.748 (*n =* 47); international standards NIST S1, δ^34^S average value 0.20‰ (accepted value 0.30‰), standard deviation 0.40 (*n =* 20); NIST S2, δ^34^S average value 22.28‰ (accepted value 22.62‰), standard deviation 0.65 (*n =* 19); and NIST S3, δ^34^S average value 32.54‰ (accepted value 32.49‰), standard deviation 0.76 (*n =* 17). Laboratory standards were repeated with every 10 samples and were used to correct for linearity and instrument drift over a 72 h analytical run. The isotope ratios are expressed in the delta (δ) notation in parts per million (‰): δ*X* = [(*R*_sample_/*R*_standard_) − 1], where *X* = ^34^S and *R* = the ratio of ^34^S/^32^S isotopes in a given sample compared with V‐CDT (Vienna – Canyon Diablo Troilite).

### 2.2 Estimating tail hair growth rate

We calculated tail hair growth rate in order to estimate the location of each individual wildebeest at the time the tail hair sample was growing. To calculate growth rate of a tail hair, we divided the total length of a tail hair for each individual by the number of days that a hair grew (i.e. the difference between collaring and recapture dates). To assess whether older hair might fragment at a faster rate than younger hair (Figure S2), we tested if the growth rate was different between individuals whose hair grew for longer than 13 months (i.e. 395 days) against the ones whose hair grew for shorter than this time using a generalised linear model. We also calculated what proportion of tail hair growth period occurs during wet and dry seasons as well as during the lactation period (Table 1) (note that wildebeest reproduction is highly synchronous with calving in February and weaning in September, which enables us to estimate the lactation period for each animal). We included this information in the generalised linear model to test if tail hair growth rate differed by season and reproductive status.

**TABLE 1:** Net tail hair growth rate per day for each individual wildebeest and a mean growth rate for all 11 GPS‐collared wildebeest.

ID	Start date	End date	No. segments	Tail length (mm)	Growth days	Days dry	Days wet	Days lactating	Net growth rate (mm/day)
WB_417	08/06/2013	02/07/2014	25	200	389	135	254	120	0.514
Wb_418	09/06/2013	03/07/2014	23	184	389	150	239	0	0.473
WB_419	08/06/2013	05/07/2014	24	192	392	144	248	120	0.489
WB_420	10/06/2013	06/07/2014	26	208	391	165	226	120	0.531
WB_422	10/06/2013	06/07/2014	21	168	391	180	211	120	0.429
WB_551	26/05/2016	30/11/2017	37	296	553	345	208	240	0.535
WB_552	26/05/2016	30/11/2017	39	312	553	345	208	0	0.564
WB_553	26/05/2016	29/11/2017	33	264	552	345	207	240	0.478
WB_578	24/03/2018	23/06/2019	36	288	456	210	246	120	0.631
WB_580	27/04/2017	24/06/2019	40	320	788	210	578	120	0.406
WB_582	24/03/2018	25/06/2019	33	264	458	195	263	0	0.576
**Net growth rate (mm/day) mean:**									**0.511**
**SD:**									**0.062**

### 2.3 Establishing the lag time for δ^34^S absorption in tail hair for migratory wildebeest

To establish the lag time for δ^34^S absorption between ingestion and deposition in tail hair of migratory wildebeest (*N* = 5), we georeferenced each segment of the tail hair (*N* = 118), conditional on an assumed wildebeest specific growth rate, and extracted the corresponding mean δ^34^S isotope value from the Serengeti sulfur isoscape.^6^ The growth rate for each wildebeest was taken from a normal distribution parameterised by the mean and standard deviation of the observed net growth rate. We extracted the δ^34^S values for every GPS point during the period of growth of each section of tail hair to estimate the mean value for the 8 mm section as a whole. We then fitted a linear regression in which the slope was fixed to be one between the isotope value of the tail hair segment against the corresponding mean isotope value from the isoscape at lags ranging from 0 to 160 days at 10‐day intervals. We used the *r‐*squared metric from each regression to determine the lag that generated the best fitting regression model. We repeated this process 5000 times (bootstrapping the individual wildebeest tail hair growth rates) to generate the mean and 95th percentile intervals (PIs) on the estimated lag time.

## 3 RESULTS

### 3.1 Tail hair growth rate

Individual net rate of growth of tail hair from GPS‐collared wildebeest varied between 0.40 and 0.63 mm per day (mean = 0.511, SD = 0.062) (Table 1). We did not observe any significant effect of either season (coefficient = −0.178, *t =* 0.819, *p =* 0.439) or reproductive status (coefficient = −0.077, *t =* 0.544, *p =* 0.603) on the wildebeest net tail hair growth rate. There was no evidence that net growth rate of hair that grew for more than 13 months was different from that of hair that grew for less than 13 months (coefficient = 0.022, *t =* 0.049, *p =* 0.662), suggesting that net growth rate remains relatively constant. However, we note that if the distal end of the tail hair is fragmenting (i.e. eroding; see Figure S2) independently of the age of the hair, then our estimated net growth rate is likely to be lower than the true growth rate.

### 3.2 Estimates of lag time and baseline fractionation factor for δ^34^S in tail hair for migratory wildebeest

**FIGURE 3:**
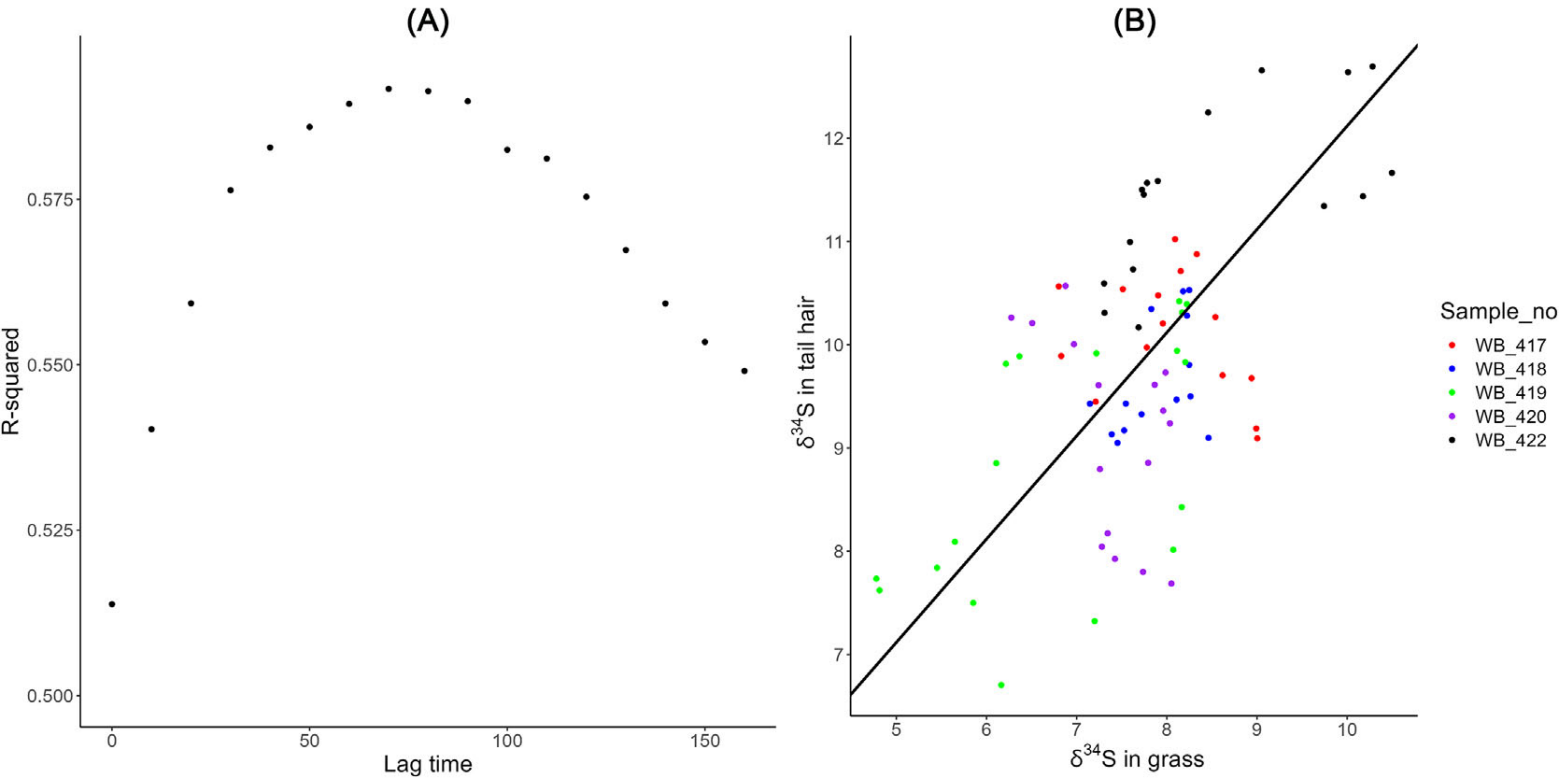
(A) *r*‐squared values from regression models relating δ^34^S in tail with georeferenced δ^34^S isoscape values at different time lags and (B) the regression plot of our optimum 78 day lagged model (including individual as a random effect indicates an individual level standard deviation on discrimination factor of ~0.8). [Color figure can be viewed at wileyonlinelibrary.com]

The best fitting regression model between δ^34^S in the tail hair and δ^34^S on the isoscape was found assuming a lag time of 78 days (95th PI 60–110 days; Figure 3A) and a baseline fractionation factor of 2.118‰ (95th PI 2.000–2.156‰; Figure 3B). The lag estimate is insensitive to lower tail hair growth rates but increases by about 10 days for every unit standard deviation (0.062) tail hair growth rate is increased by. Slopes of more or less than one can be imposed on the analysis, generating fits with equivalently well‐fitting models, and slightly different lag times (e.g. a slope of 0.75 generates lower lags of around 60 days, and 1.25 generates higher lags of around 90 days). Fitting both the intercept and slope results in very marginally better fitting model and a lower lag estimate of closer to 40 days but an unrealistically high discrimination factor of about 5.

## 4 DISCUSSION

The primary result from this study suggests the delay between ingestion and deposition of δ^34^S isotopes in the tail hair is substantial and here estimated to be about 78 days. This suggests that sulfur in the animal's body passes through two or more metabolic processes before being deposited in the tail hair. These findings are important because understanding how metabolic delays and processing speed influence the variation of δ^34^S in biological material such as hair has important implications for making inferences about animal movements or dietary changes. Secondary results indicate that wildebeest tail hair grows at a constant rate invariant of season and pregnancy status, and there is no evidence of differential distal fraying or disintegration, and that tail hair length maps straightforwardly onto time.

There are several metabolic processes in the body that use sulfur and which may account for the long lags we observed between ingestion and deposition of ^34^S in tail hair as indicated by our results. For example, Fry and Arnold^37^ suggested isotopic turnover to be a result of two processes: tissue growth and catabolic turnover. The slow δ^34^S turnover rate we observe could represent the fact that S is not directly involved in the process of obtaining metabolic energy, unlike C and N.^38^ For example, sulfur in the hair is primarily derived from cysteine and methionine.^29^, ^39^ Methionine is a nutritionally indispensable amino acid that is usually acquired entirely from dietary sources.^40^ Methionine is also used as a precursor for cysteine, which is a nonessential amino acid (i.e. it is synthesised from other amino acids).^40^ For ruminants, methionine might not be an entirely nutritionally indispensable amino acid because rumen microbes can synthesise methionine.^41^ However, supplementing ruminants' diet with the S‐containing diet has proven to improve the metabolism of amino acids.^42^ Therefore, the observed levels of δ^34^S in the tail hair are likely to result from a combination of recent food intake and turnover of proteins in tissues derived from foods that were consumed over longer time scales, and observed lag times may reflect a balance in the preponderance of these two processes. Indeed, it is likely that the lag is better represented by a form of weighted distribution than a single value. We attempted to estimate such distributions using a variety of different approaches that generated a large number of alternative distributed lag models, but while we found many that fitted the data nearly as well as a single fixed lag time, we did not find one that fitted the data better.

The isotopic turnover rate not only is a function of the metabolic processes and tissues but also scales with body mass^29^, ^43^ which may compromise the utility of S isotopes for geolocating large animals. Small animals such as mice^44^ integrate isotope signals over a short period of time compared with large animals.^45^ For instance, Bahar et al^38^ reported the turnover rate of δ^34^S in the longissimus muscle of beef cattle is in excess of 219 days (approximate weight: 500 kg), while our study estimates 78 days for δ^34^S in wildebeest tail hair (approximate weight: 160 kg) suggesting that the turnover rate of δ^34^S in small ungulates may be faster than in large ungulates. Therefore, using δ^34^S in tail hair to study animal movement may only be useful in small‐ to medium‐sized animals with relatively long hair but may not be applicable for animals with short hair or very large herbivores such as elephant.

The rate at which δ^34^S is processed in herbivores may also be a function of diet quality which itself is likely to vary seasonally. For instance, forage with high protein content such as C_3_ forbs is processed differently from forage with low protein such as C_4_ grass.^46^ Variations in dietary protein also alter the isotopic discrimination^2^, ^16^ between different tissues within the body such as muscle, blood or skeletal tissue.^47^ For example, Richards et al^2^ switched the diet of two horses from their long‐term ^34^S‐rich diet (δ^34^S = 10.8‰) to a ^34^S‐poor diet (δ^34^S = −1.9‰) for a period of 21 weeks, before switching back to a ^34^S‐rich diet (δ^34^S = 10.5‰) for a further 19 weeks. Tail hair was collected from each individual and analysed for δ^34^S. They noted that the C_3_ and C_4_ diets with which they supplied the horses were isonitrogenous but had different protein content with the C_3_‐based feed having higher protein content than the C_4_‐based one. The authors reported a larger diet–hair fractionation when horses were fed the protein‐poor C_4_‐based feed but lower fractionation levels when fed with C_3_ hays. The lower digestible protein in the C_4_ feed could be associated with increased recycling of body proteins constructed while on the C_3_ feed. Perhaps the time lag we report for wildebeest could also be controlled by diet type. For example, individuals who feed on a relatively similar diet in the same area for a relatively long time, such as cattle,^6^ may have δ^34^S values in the tail hair that accurately reflect the δ^34^S of their diet (Figure S3). However, wildebeest have been reported to be mostly grazers, feeding on C_4_ grass,^48^, ^49^, ^50^ but have also been reported to supplement their diet with C_3_ plants,^51^ providing further support for the idea of a distributed lag time.

The spatial variation of forage quality is a function of soil properties such as parent material or cation exchange capacity^46^; however, parent material also determines δ^34^S.^2^ Therefore, in areas with diverse parent material such as Serengeti, the protein content of the forage may be correlated with δ^34^S isotopes^52^ (Figure S4). In these instances, using δ^34^S to make ecological inferences about animal movement may be complicated by the quality of the diet as well as collinearities between forage protein and δ^34^S.

## 5 CONCLUSION AND FUTURE STUDIES

Our analysis demonstrates the underlying complexities when using δ^34^S to estimate animal movement. These complexities are likely caused by a mixture of animal physiology (metabolism) and diet quality. Since wildebeest are eating a mixture of both low‐ and high‐protein diets seasonally as they migrate between areas of high and low δ^34^S, the δ^34^S deposited in the hair likely represents an averaged value. Therefore, δ^34^S in the tail is a challenging approach for geolocation of wildebeest because of the long time lags between δ^34^S ingestion and deposition, lags that may also depend on changing forage quality over time. However, if animals were moving across an S isoscape but were on a single diet with stable protein concentrations then these challenges may be overcome. A possible avenue for future studies might be to explore the contribution of δ^34^S as incorporated from diet only. The δ^34^S from diet may be a truer reflection of δ^34^S in the landscape. Currently, the δ^34^S we observe in the tail hair of wildebeest is composed of both essential (from diet) and nonessential amino acids that are embedded within forage of different protein concentrations which complicates the applicability of using δ^34^S as a geolocator for fast moving migratory animals. This calls for more controlled diet studies in which we can establish the influence and timing of dietary shifts for both wild and domesticated ungulates to ascertain this information. Furthermore, an understanding of the timing of dietary shift in wildebeest might also help to improve the estimates of our lag time, particularly by identifying the patterns of seasonal variation in isotopic discrimination values between grass and hair due to changes in food quantity, food quality or energy use.

## AUTHOR CONTRIBUTIONS

**Zabibu Kabalika:** Conceptualization; data curation; formal analysis; funding acquisition; investigation; methodology; writing—original draft; writing—review and editing. **Daniel T Haydon:** Conceptualization; formal analysis; methodology; supervision; writing—original draft; writing—review and editing. **Rona A.R. McGill:** Conceptualization; data curation; supervision; writing—original draft; writing—review and editing. **Juan M Morales:** Conceptualization; formal analysis; methodology; writing—original draft. **Thomas A Morrison:** Conceptualization; methodology; supervision; writing—original draft. **Jason Newton:** Conceptualization; data curation; supervision; writing—original draft; writing—review and editing. **Grant Hopcraft:** Conceptualization; formal analysis; funding acquisition; methodology; supervision; writing—original draft; writing—review and editing.

## CONFLICT OF INTEREST STATEMENT

The authors declare no conflict of interests.

### PEER REVIEW

The peer review history for this article is available at https://www.webofscience.com/api/gateway/wos/peer-review/10.1002/rcm.9674.

## Supporting information


**Figure S1:** Variation of δ^34^S across length of a tail hair for the migrant and resident GPS collared wildebeest from the Serengeti ecosystem. The figure depicts that, migrant wildebeest have a cyclic variation of their δ^34^S (likely reflecting a migratory cycle) compared to residents who have a random variation (data source: Kabalika et al – unpublished data).

**Figure S2:** Microscopic view of a tail hair structure for root and tip ends showing that the hair root is thicker and more stable compared to the tip which is thinner and fragile, suggesting that the hair tip erodes as it grows. a) is a representative tail hair strand for W580 which grew for longer than 13 months and b) is a representative tail hair strand for W422 which grew for less than 13 months (Data source: Personal observation).

**Figure S3:** Relationship between δ^34^S values in grass and those in the tail hair of the resident wildebeest from the Serengeti, suggesting that the δ^34^S values of the individuals who feed over a relatively long time in the same area accurately reflect the δ^34^S of their diet (data source: Kabalika et al – unpublished data). Please note that, the resident wildebeest presented in this graph are only from western corridor. This is because, the δ^34^S values in the tail hair for the mara residents could not be paired with the δ^34^S of the isoscape as they appear outside the range of our predicted δ^34^S isoscape (Refer figure 2b). Including individual as a random effect indicates an individual level standard deviation on discrimination factor of ~ 0.276.

**Figure S4:** Relationship between S and N concentrations in grass from the Serengeti ecosystem, suggesting that S is correlated with the protein content of the grass. The units are in concentration per unit mg of grass (data source: Kabalika et al – unpublished data).

## Data Availability

Upon acceptance for publication, all data that support the findings of this study will be publicly availble at the university of glasgow's data repository.
