# Supplementary figures and images for: Using sulfur stable isotope ratios (δ34S) for animal geolocation: Estimating the delay mechanisms between diet ingestion and isotope incorporation in tail hair
Source: Rapid Commun Mass Spectrom. 2023 Nov 28;38(2):e9674. doi: 10.1002/rcm.9674 (PMC10909487; doi:10.1002/rcm.9674)

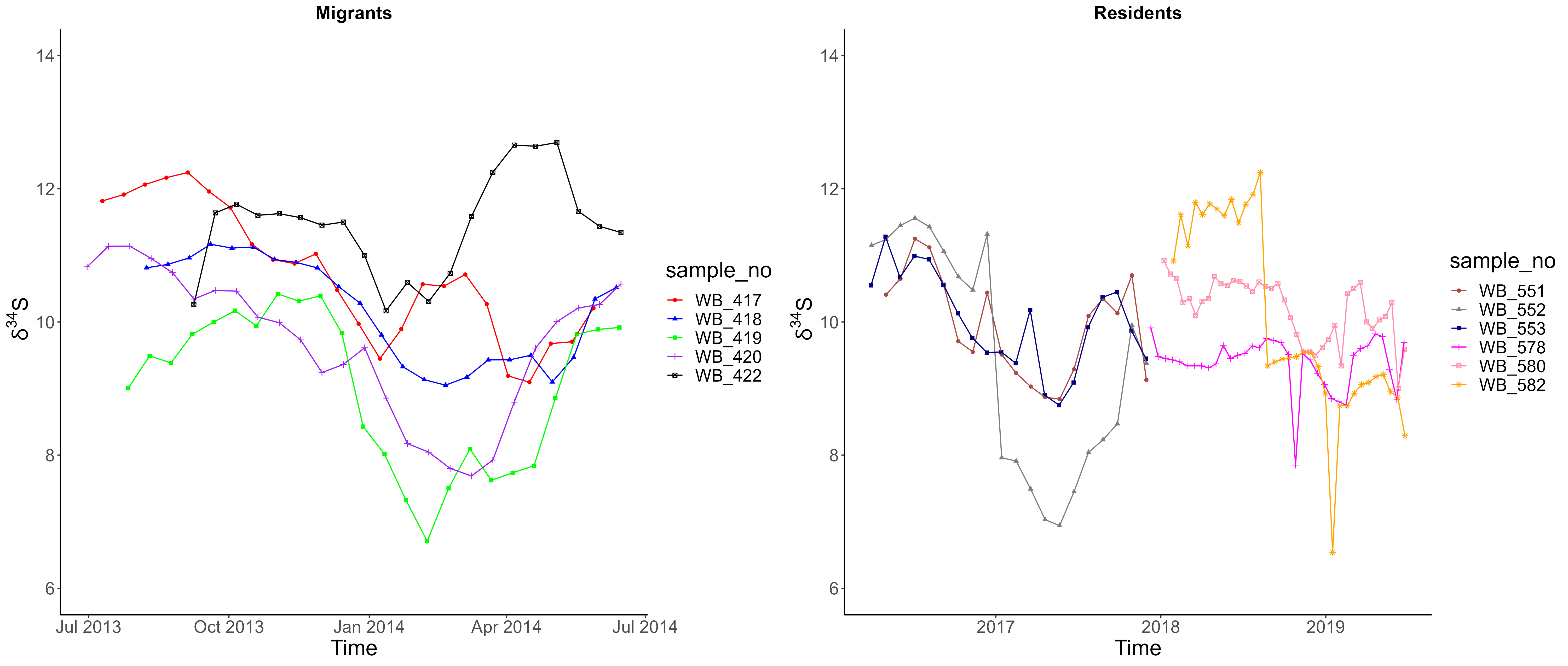

Supplement: Supplementary file 1 — Figure S1: Variation of δ34S across length of a tail hair for the migrant and resident GPS collared wildebeest from the Serengeti ecosystem. The figure depicts that, migrant wildebeest have a cyclic variation of their δ34S (likely reflecting a migratory cycle) compared to residents who have a random variation (data source: Kabalika et al – unpublished data). [file RCM-38-e9674-s004.png]

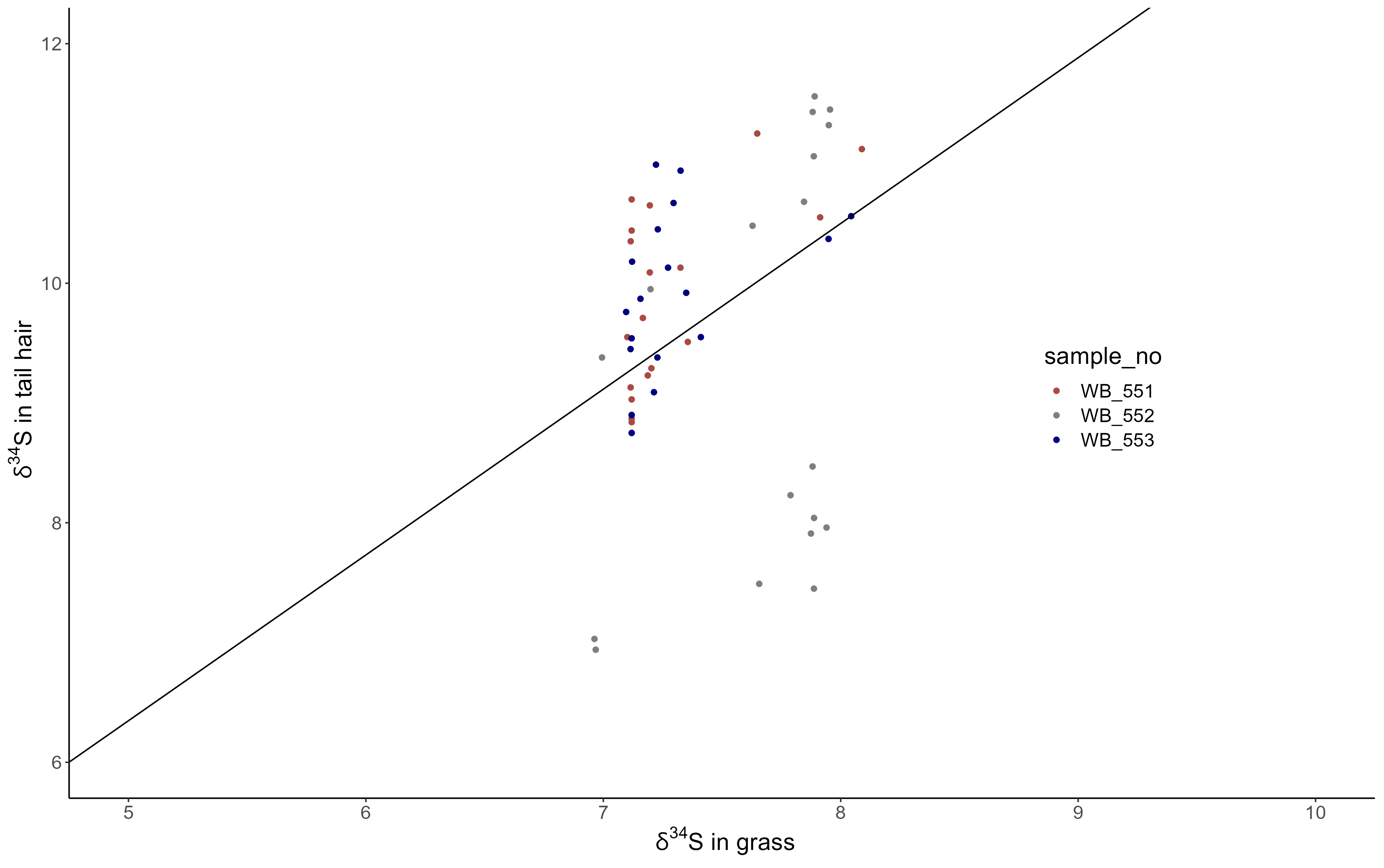

Supplement: Supplementary file 3 — Figure S3: Relationship between δ34S values in grass and those in the tail hair of the resident wildebeest from the Serengeti, suggesting that the δ34S values of the individuals who feed over a relatively long time in the same area accurately reflect the δ34S of their diet (data source: Kabalika et al – unpublished data). Please note that, the resident wildebeest presented in this graph are only from western corridor. This is because, the δ34S values in the tail hair for the mara residents could not be paired with the δ34S of the isoscape as they appear outside the range of our predicted δ34S isoscape (Refer figure 2b). Including individual as a random effect indicates an individual level standard deviation on discrimination factor of ~ 0.276. [file RCM-38-e9674-s003.png]

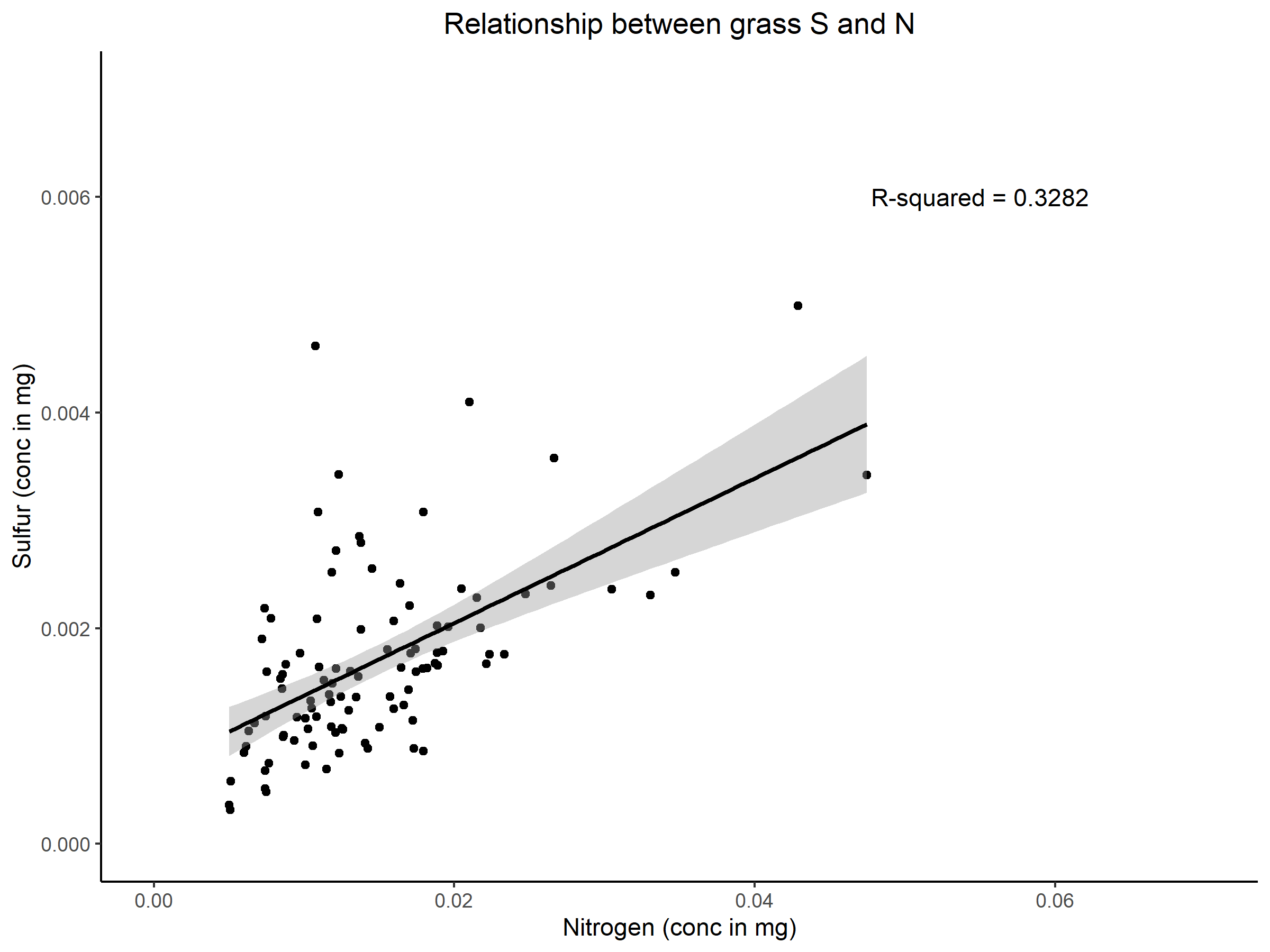

Supplement: Supplementary file 4 — Figure S4: Relationship between S and N concentrations in grass from the Serengeti ecosystem, suggesting that S is correlated with the protein content of the grass. The units are in concentration per unit mg of grass (data source: Kabalika et al – unpublished data). [file RCM-38-e9674-s002.png]
