# Supplementary material for: Using sulfur stable isotope ratios (δ34S) for animal geolocation: Estimating the delay mechanisms between diet ingestion and isotope incorporation in tail hair
Source: Rapid Commun Mass Spectrom. 2023 Nov 28;38(2):e9674. doi: 10.1002/rcm.9674 (PMC10909487; doi:10.1002/rcm.9674)
Supplement: Supplementary file 2 — Figure S2: Microscopic view of a tail hair structure for root and tip ends showing that the hair root is thicker and more stable compared to the tip which is thinner and fragile, suggesting that the hair tip erodes as it grows. a) is a representative tail hair strand for W580 which grew for longer than 13 months and b) is a representative tail hair strand for W422 which grew for less than 13 months (Data source: Personal observation). [file RCM-38-e9674-s001.docx]

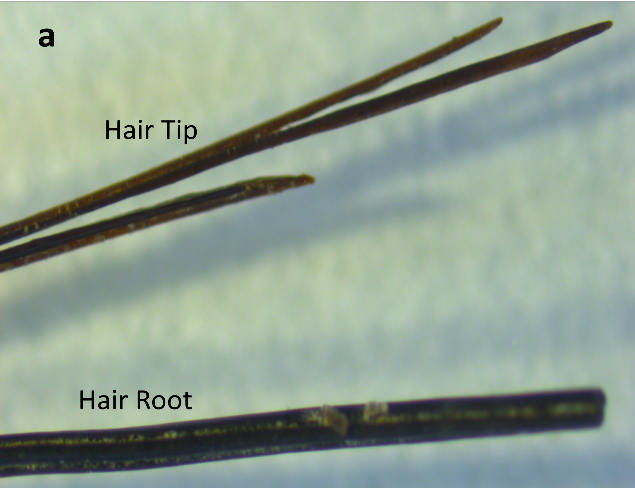

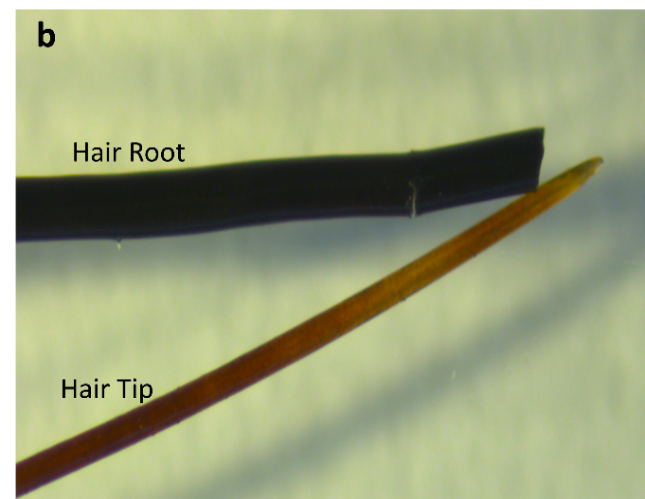


Figure S2: Microscopic view of a tail hair structure for root and tip ends showing that the hair root is thicker and more stable compared to the tip which is thinner and fragile, suggesting that the hair tip erodes as it grows. a) is a representative tail hair strand for W580 which grew for longer than 13 months and b) is a representative tail hair strand for W422 which grew for less than 13 months (Data source: Personal observation).
